# Protein surface shielding agents in protein crystallization

**DOI:** 10.1107/S0909049510032450

**Published:** 2010-11-05

**Authors:** J. Hašek

**Affiliations:** a Institute of Macromolecular Chemistry, Academy of Sciences of the Czech Republic, Czech Republic

**Keywords:** protein adhesion, protein surface shielding agents, intermolecular interactions, competitive adhesion modes, polymer crystallization screens

## Abstract

The molecules adhering temporarily on the surface of protein molecules change the propensity of protein molecules to deposit on the crystal surface in a definite position and orientation. The concepts of *competitive adhesion modes* and *protein surface shielding agents* acting on the surface of molecules in a non-equilibrium process of protein crystallization provide a useful platform for the control of crystallization. The desirable goal, *i.e.* a transient preference of a single dominating adhesion mode between protein molecules during crystallization, leads to uniform deposition of proteins in a crystal. This condition is the most important factor for diffraction quality and thus also for the accuracy of protein structure determination. The presented hypothesis is a generalization of the experimentally well proven behaviour of hydrophilic polymers on the surface of protein molecules of other compounds.

## Abstract 2

The crystallization process can be controlled by protein surface shielding agents blocking undesirable competitive adhesion modes during non-equilibrium processes of deposition of protein molecules on the surface of growing crystalline blocks. The hypothesis is based on a number of experimental proofs from diffraction experiments and also retrieved from the Protein Data Bank.

## 1. Introduction

In the last 20 years crystallization methods have developed into an efficient tool for protein crystal production (*e.g.* Ducruix & Giege, 1992 ▶; Bergfors, 1999 ▶; McPherson, 1999 ▶). However, the quality of protein structures deposited in the Protein Data Bank (PDB) is still far behind the desirable standard. In spite of the fact that a resolution of better than 1.5 Å is usually necessary for a reliable interpretation of the processes taking place in biomolecular systems, more than 50% of deposited structures in the PDB have a diffraction limit (resolution) worse than 2.0 Å. It is often supposed that a lower diffraction quality of biomacromolecular crystals is caused by a high content of water in the crystalline state and by high conformational flexibility. Of course, the primary reasons for low diffraction quality are more complex. This can be seen, for example, in the structure of laccase with clear final map of electron density in spite of the fact that it contains four times more water than the protein itself (PDB code 3cg8, water contents 81%) (Skálová *et al.*, 2009 ▶).

## 2. Crystallization control

The principal reason for low diffraction quality of many protein crystals is not the high content of water itself but the fact that proteins have a large surface area with more energetically favourable adhesion areas allowing deposition of some molecules in different orientations than others. Deposition errors sum on long distance disturbing thus the long-range periodicity and consequently also the diffraction quality of the crystal. High water content in protein crystals helps these processes, allowing for ‘local plasticity’ which helps to decrease the energy demands evoked by stacking faults during molecular deposition in the growing crystals. It smoothly explains the existence of so-called ‘phantom crystals’, optically nice crystals without any diffraction, frequently reported in many protein laboratories.

*A posteriori* analysis of experimentally verified intermolecular contacts in different crystal forms (Hašek, 2006 ▶) helped us to understand the intermolecular adhesion playing an important role in the crystallization processes. The analysis showed that proteins usually have many adhesion modes, some of them being mutually compatible in a single-crystal form, and others not (Hašek, 2006 ▶). The theory of *the crystallographically compatible adhesion modes* says that well diffracting crystals grow only in cases where the crystallization process is controlled by a single *dominating adhesion mode* (DAM), *i.e.* when there is temporally only one preferred adhesion mode between two macromolecules realised in the crystallization buffer around the growing crystal.

When the crystallization proceeds slowly, then a majority of macromolecules deposit on the surface of the growing crystalline nuclei according to this single adhesion mode. A low number of macromolecules depositing randomly in different crystallographically incompatible adhesion modes remain with high probability alone on the growing surface, and without three-dimensional fixation in the crystal block quickly dissolve again. Thus, the crystal growth driven by a single DAM proceeds regularly without crystallographically significant errors.

In the case where the protein dissolved in a given crystallization buffer has more *competitive adhesion modes* (CAMs), then compact molecular islands belonging to *crystallographically incompatible adhesion modes* are formed on the growing crystal surfaces. Molecules in the larger molecular islands are already well stabilized in the growing nuclei and thus they do not dissolve easily. The growing crystal has in this case a number of stacking faults, and loses long-range periodicity necessary for good diffraction.

Appreciating this, we can interfere in the crystallization process using relatively simple tools. Using middle-sized molecules temporarily adhering with different affinity on crystallographically important areas on a protein surface one can largely modify the kinetics of adhesion between protein molecules. These non-protein molecules, evoking for this moment very specific adhesion between protein molecules, are called *protein surface shielding agents* (PSSAs).

Good PSSAs should not be harmful to protein stability and should adhere selectively to crystallographically important areas on the protein surface. The PSSA–protein adhesion should be strong enough to protect the protein–protein adhesion in unwanted adhesion modes during crystallization. However, it must be simultaneously low enough because the adhering PSSA should be easily expelled from the crystal surface by the protein–protein adhesion of new protein molecules depositing on the crystal surface in the non-equilibrium process of the crystal growth.

Many molecules adhering temporarily on crystallo­graphically important areas on the protein surface may act as PSSAs. Thus, many low-molecular and macromolecular PSSAs have already been used intuitively in crystallization screens, intended for different reasons and described as precipitants, additives or cryoprotectants as a rule.

It appears that polymeric molecules have a special position and have promising properties as PSSAs. In particular, the hydrophilic polymers possessing a specific affinity to the protein surface used in ∼20000 protein structure determinations, and analysed by Hašek (2006 ▶), deserve special attention because they form a large exclusion volume around the crystallographically significant adhesion area on the protein surface and thus exhibit stronger effects in comparison with most of their low molecular equivalents.

## 3. Polymers and co-polymers adhering on protein surface

The structure database of polymers, showing the polymer structure in the crystalline state, is described by Hašek & Labský (1995 ▶). However, hydrophilic polymers only can serve as efficient PSSAs. The behaviour of hydrophilic polymers of polyethyleneoxide type in protein systems and the protein–polymer interactions have been discussed by Hašek (2006 ▶).

Polyethyleneoxide (often called polyethylene glycol with the abbreviation PEGxxx, where xxx denotes the average molecular weight of the polymer) has been largely used as a protein precipitant. In particular, PEG550, PEG1400, PEG3500 and PEG8000 have been used for a long time in large concentrations (10–45%) as principal precipitation components in many commercial crystallization screens. These polymers form semi-stable (calculated enthalphy in tens of kJ mol^−1^) non-covalent multiple ion-dipole bonds to positively charged residues Lys, Arg and His. Owing to their large molecular weight they form large exclusion volumes selectively around selected positive charges exposed on the protein surface, thus changing the preference of different adhesion modes between protein molecules. The PSSA hypothesis (Hašek, 2006 ▶) is in full agreement with many static experiments. No kinetic experiments of crystallization processes under different conditions are planned by our group.

We deduce that the success of the crystallization screens with polyethyleneglycol precipitants and their high popularity can be rationalized by the fact that, parallel to their precipitation effect, they simultaneously operate as efficient PSSAs (Hašek, 2006 ▶). However, none of the screens was designed considering precipitation and the PSSA effects separately. Only two polymer crystallization screens, PolyA and PolyB described by Skálová *et al.* (2010 ▶), were based on the supposed action of PSSAs, on the commercial availability of the co-polymers and on the non-toxicity of the co-polymers. Thus, new-generation crystallization screens allowing a deeper control of the crystallization process may be expected in the near future.

## 4. Conclusions

The concept of different adhesion modes mutually competing during the crystallization process is an experimentally well proven hypothesis, namely for PSSAs of polyethyleneglycol type.

(i) It is based on an experimentally well proven selective binding of the PEG-type polymers on the protein surface at areas critical for crystal growth (several hundred structures in the PDB).

(ii) The parallel PSSA effect of polymer precipitants explains the commercial success and popularity of crystallization screens based on the PEG-type polymer precipitants.

(iii) It offers a simple alternative way and explanation of the success of methods for improving the crystallization process by ‘lysine methylation’ (Walter *et al.*, 2006 ▶) and by ‘surface entropy reduction’ by mutation of ‘residues with a high entropy content’ (Goldschmidt *et al.*, 2007 ▶).

(iv) It provides a simple and smooth explanation of many other phenomena observed in protein crystallization, for example a change of the space group of crystals owing to tiny changes in buffer, *etc.*

(v) It gives a very natural explanation for so-called phantom crystals, *i.e.* optically well looking crystals without any diffraction, and offers ways to improve the diffraction quality of crystals.

PSSAs, *i.e.* small- and medium-sized molecules with moderate adhesion to specific areas on the molecular surface, are in some cases critical for the kinetics of crystal growth. Proper PSSAs can diminish a frequency of stacking faults leading to crystals of better diffraction quality by blocking the detrimental adhesion modes between the protein molecules. The PSSA concept alone does not offer a direct instrument for the prediction of the optimal PSSA for crystallization of a new protein of unknown structure. Thus, the procedure still involves some trial-and-error mechanisms. However, it provides a better understanding of the crystallization process and a clearer background for the design of crystallization experiments. The new concepts discussed here can help in the control of crystallization processes and the development of universal PSSAs with well predictable effects.
